# Ubiquitylation-dependent regulation of NEIL1 by Mule and TRIM26 is required for the cellular DNA damage response

**DOI:** 10.1093/nar/gkw959

**Published:** 2016-10-18

**Authors:** Matthew J. Edmonds, Rachel J. Carter, Catherine M. Nickson, Sarah C. Williams, Jason L. Parsons

**Affiliations:** Cancer Research Centre, Department of Molecular and Clinical Cancer Medicine, University of Liverpool, 200 London Road, Liverpool L3 9TA, UK

## Abstract

Endonuclease VIII-like protein 1 (NEIL1) is a DNA glycosylase involved in initiating the base excision repair pathway, the major cellular mechanism for repairing DNA base damage. Here, we have purified the major E3 ubiquitin ligases from human cells responsible for regulation of NEIL1 by ubiquitylation. Interestingly, we have identified two enzymes that catalyse NEIL1 polyubiquitylation, Mcl-1 ubiquitin ligase E3 (Mule) and tripartite motif 26 (TRIM26). We demonstrate that these enzymes are capable of polyubiquitylating NEIL1 *in vitro*, and that both catalyse ubiquitylation of NEIL1 within the same C-terminal lysine residues. An siRNA-mediated knockdown of Mule or TRIM26 leads to stabilisation of NEIL1, demonstrating that these enzymes are important in regulating cellular NEIL1 steady state protein levels. Similarly, a mutant NEIL1 protein lacking residues for ubiquitylation is more stable than the wild type protein *in vivo*. We also demonstrate that cellular NEIL1 protein is induced in response to ionising radiation (IR), although this occurs specifically in a Mule-dependent manner. Finally we show that stabilisation of NEIL1, particularly following TRIM26 siRNA, contributes to cellular resistance to IR. This highlights the importance of Mule and TRIM26 in maintaining steady state levels of NEIL1, but also those required for the cellular DNA damage response.

## INTRODUCTION

DNA is under constant attack by reactive oxygen species that are generated endogenously through cellular oxidative metabolism, but also by exogenous sources such as ionising radiation (IR) resulting in DNA base oxidation, base loss (apurinic/apyrimidinic or AP sites) and DNA strand breaks. Indeed, ∼10 000 DNA base damage events are estimated to occur in every human cell per day as a consequence of cellular metabolism (1), which if left unrepaired, can cause genome instability, drive mutagenesis and have been implicated in the development of premature ageing, neurodegenerative diseases and cancer. To combat this DNA damage onslaught, the base excision repair (BER) pathway has evolved as the major cellular mechanism for excising damaged DNA bases and replacing these with the correct undamaged nucleotides (2). BER is achieved in a co-ordinated manner by a specific subset of enzymes and is firstly initiated by the removal of damaged DNA bases by DNA glycosylases, of which 11 human enzymes are known to exist, each with their own substrate specificity (3). The DNA glycosylases can be divided into two classes depending on their mode of action. A monofunctional enzyme removes the damaged base creating an AP site which is recognised by AP endonuclease-1 (APE1). Alternatively a bifunctional DNA glycosylase, in addition to removing the damaged base, will also incise the DNA backbone creating a single nucleotide gap containing either a 3′-α, β-unsaturated aldehyde or a 3′-phosphate that requires processing by APE1 or polynucleotide kinase phosphatase (PNKP), respectively. DNA polymerase β (Pol β) then inserts the correct nucleotide into the repair gap and DNA ligase IIIα-X-ray cross complementing protein 1 (Lig IIIα-XRCC1) complex seals the DNA ends.

The endonuclease VIII-like protein 1 (NEIL1), a bifunctional DNA glycosylase, was uncovered over a decade ago and was initially thought to be a backup repair enzyme for 8-oxoguanine DNA glycosylase (OGG1) and endonuclease III homologue (NTH1) (4–6). This was due to the fact that it shares a similar substrate specificity to both of these enzymes in cleaving oxidised DNA bases. However, since its discovery, there has been increasing biological evidence that NEIL1 is involved in the repair of specific types of DNA base damage. These include DNA base damage in close proximity to each other which is frequently induced by IR (7,8) and base damage from telomeric DNA (9,10). NEIL1 has also been shown to bind to interstrand cross-links (11). Furthermore, NEIL1 excises lesions both from bubble structures and single stranded DNA mimicking DNA replication and transcription sites (12,13). Further data have also implicated the involvement of NEIL1 specifically in a replication-associated DNA repair role (14). The importance of NEIL1 in the cellular response to DNA damage has been shown by the observation that NEIL1 protects cells from hydrogen peroxide and IR-induced cell death (15,16). Interestingly, a deficiency of NEIL1 in knockout mouse models displays an obese phenotype associated with a metabolic syndrome (17,18). NEIL1 also appears to play a role in reducing brain damage following ischemic stroke (19) and loss of NEIL1 causes defects in olfactory function in mice, which are the earliest symptoms of age-related neurodegenerative disorders such as Alzheimer's and Parkinson's diseases in humans (20). Indeed, reduced NEIL1 protein levels have been discovered in tissue extracts from Alzheimer's patients (21). Taken together, these data suggest that NEIL1 is not just a backup system for NTH1 and OGG1, but that it plays an important cellular role and that its cellular steady state levels should be accurately maintained. However the molecular mechanisms controlling NEIL1 protein levels are currently unknown.

The ubiquitin proteasome pathway (UPP) is an important mechanism for controlling cellular protein levels involved in several cellular processes, including DNA repair (22). This pathway involves the attachment of ubiquitin moieties onto specific lysine residues within the target protein, which is ultimately performed by E3 ubiquitin ligases, of which >600 enzymes exist in the human genome, each with their own specific targets. If multiple ubiquitin chains are added, termed polyubiquitylation, the target protein is usually degraded by the proteasome. The UPP driven by specific E3 ubiquitin ligases has been discovered to be crucial in regulating steady state and DNA damage-induced levels of BER proteins, and ultimately cellular BER capacity (23,24). However, the enzymes specifically catalysing ubiquitylation-dependent degradation of NEIL1, and therefore the mechanisms controlling the cellular levels of this key DNA repair protein, have not yet been identified. Here, we report the identification of two E3 ubiquitin ligases, Mcl-1 ubiquitin ligase E3 (Mule; also known as ARF-BP1 and HUWE1) and tripartite motif 26 (TRIM26) as the major cellular enzymes involved in the polyubiquitylation, and subsequent degradation, of NEIL1 both *in vitro* and *in vivo*. As a consequence of this, we demonstrate that these enzymes play crucial but distinct roles in controlling cellular protein levels of NEIL1 required for the response to DNA damage.

## MATERIALS AND METHODS

### Materials

NEIL1 antibodies were kindly provided by Dr T. Rosenquist. TRIM26 (ab89290) and fibrillarin (ab4566) antibodies were from Abcam (Cambridge, UK). His-tag antibodies were from Millipore (Watford, UK). Tubulin and actin antibodies were from Sigma-Aldrich (Gillingham, UK). HeLa cell pellets for protein fractionation by column chromatography were from Cilbiotech (Mons, Belgium). Ubiquitin, E1 and E2 enzymes were purchased from Boston Biochemicals (Cambridge, USA). The mammalian expression plasmid for TRIM26 and the bacterial expression plasmid for NEIL1 were kindly provided by Prof A. Garcia-Sastre and Prof. S. Wallace, respectively. The bacterial expression plasmid for truncated Mule containing the E3 ligase HECT domain was kindly provided by Prof. M. Eilers. The full length cDNA for *TRIM26* was recloned into pET28a by ligation-independent cloning (25). Full length *NEIL1* cDNA was also recloned using the same approach into pCMV-Tag3a vector for mammalian expression. Site-directed PCR mutagenesis was used to generate site-specific mutants within NEIL1. Primers were designed using SDM-Assist (26) and also contain ‘silent’ restriction sites for screening of clones. These are listed in the Supplementary Data (Supplementary Table). His-tagged TRIM26 and NEIL1 were overexpressed in Rosetta2(DE3)pLysS bacterial cells (Merck-Millipore, Watford, UK) and purified using HisTrap column chromatography (GE Healthcare, Little Chalfont, UK). TRIM26 was additionally purified using ion exchange (Mono Q 5/5 GL) chromatography (GE Healthcare, Little Chalfont, UK). GST-tagged truncated Mule was also overexpressed in Rosetta2 (DE3)pLysS bacterial cells but purified by GST-Trap column chromatography, followed by Superdex 75 size exclusion chromatography (GE Healthcare, Little Chalfont, UK).

### RNA interference and cell irradiation

U2OS cells were cultured in Dulbecco's modified Eagle's medium (DMEM) supplemented with 10% fetal bovine serum, 2 mM l-glutamine, 1× penicillin-streptomycin and 1× non-essential amino acids. All cells were cultured at 37°C in 5% CO_2_. For siRNA knockdowns, cells were grown in 10 cm dishes for 24 h to 30–50% confluence and treated with 10 μl Lipofectamine RNAiMAX transfection reagent (Life Technologies, Paisley, UK) in the presence of 200 pmol non-targeting, control siRNA (SR-CL000-005; Eurogentec, Southampton, UK), TRIM26 siRNA (5′-CCGGAGAAUUCUCAGAUAA-3′) or Mule siRNA (5′-AAUUGCUAUGUCUCUGGGACA-3′) for a further 72 h. For irradiation studies, cells were treated with X-rays using the CellRad X-ray irradiator (Faxitron, Tucson, USA) prior to processing for the various assays.

### Whole cell extract preparation and cell fractionation

Whole cell extracts were prepared from harvested cell pellets as previously described (27,28). Briefly, cell pellets were resuspended in one packed cell volume (PCV) of buffer containing 10 mM Tris–HCl (pH 7.8), 200 mM KCl, 1 μg/ml of each protease inhibitor (pepstatin, aprotinin, chymostatin and leupeptin), 100 μM PMSF and 1 mM N-ethylmaleimide (NEM). A further two PCVs of buffer containing 10 mM Tris–HCl (pH 7.8), 600 mM KCl, 40% glycerol, 2 mM EDTA, 0.2% IGEPAL CA-630, 1 μg/ml of each protease inhibitor, 100 μM PMSF and 1 mM NEM were added and the extract mixed thoroughly. The cell suspension was mixed by rotation for 30 min at 4°C, centrifuged at 40 000 rpm at 4°C for 20 min and the supernatant collected, and stored at −80°C. 40 μg protein was used for western blotting analysis. Cell fractionation was also performed as previously described (27,28). Briefly, freshly prepared cell pellets were resuspended in two PCVs of buffer containing 20 mM Tris–HCl (pH 7.8), 2.5 mM MgCl_2_, 0.5% (v/v) IGEPAL CA-630, 100 μM PMSF, 1 mM NEM and 1 μg/ml of each protease inhibitor, and incubated on ice for 10 min. Extracts were centrifuged at 10 000 rpm for 2 min at 4°C and the supernatant containing soluble proteins (S) was collected. The nuclear pellet was similarly extracted with two PCVs of buffer containing 20 mM NaPO_4_ (pH 8.0), 0.5 M NaCl, 1 mM EDTA, 0.75% (v/v) Triton X-100, 10% (v/v) glycerol, 100 μM PMSF, 1 mM NEM and 1 μg/ml of each protease inhibitor. Following centrifugation at 10 000 rpm for 2 min at 4°C, the supernatant containing chromatin bound proteins (CB) was collected. 40 μg protein from the S fraction, and the corresponding same volume of the CB fraction were used for further analysis.

### Purification of the E3 ubiquitin ligase from HeLa whole cell extracts

Whole cell extracts (prepared as described above using 20 g HeLa cell pellets) were dialysed against Buffer A (50 mM Tris–HCl (pH 8.0), 1 mM EDTA, 5% glycerol, 1 mM DTT and 100 μM PMSF) containing 150 mM KCl. The extract was clarified by centrifugation (25 000 rpm for 20 min), filtered through 0.45 μm syringe filters and then applied to a 250 ml P-11 Phosphocellulose column. The flow-through was collected (designated PC-150) and diluted 2-fold to achieve a final concentration of 75 mM KCl prior to adding to a 20 ml HiLoad Mono Q Sepharose column (GE Healthcare, Little Chalfont, UK). The column was washed with Buffer A containing 50 mM KCl and proteins eluted using a linear gradient from 50 to 1000 mM KCl. Active fractions were pooled, concentrated using Amicon Ultra-15 centrifugal filter units (Millipore, Watford, UK) and loaded onto a Superdex 200 HR 10/30 column (GE Healthcare, Little Chalfont, UK) in Buffer A containing 150 mM KCl and fractions collected. Active fractions were pooled, concentrated and buffer exchanged using Buffer B (5 mM KPO_4_ (pH 7.0), 5% glycerol, 1 mM DTT and 100 μM PMSF). The protein sample was applied to a 1 ml CHT ceramic hydroxyapatite column (Bio-Rad, Hemel Hempstead, UK) in Buffer B, and proteins were eluted using a linear gradient of 5–500 mM KPO_4_. Active fractions were pooled, diluted 10-fold in Buffer A and then loaded onto a Mono Q 5/50 GL column (GE Healthcare, Little Chalfont, UK) in buffer A containing 50 mM KCl and proteins eluted using a linear gradient of 50–1000 mM KCl. Following each chromatography stage, protein fractions were analysed for *in vitro* ubiquitylation activity employing His-NEIL1 as the substrate and active fractions pooled for the next chromatography step.

### *In vitro* ubiquitylation assay

Reactions containing 4.6 pmol His-NEIL1, 0.7 pmol GST-E1 activating enzyme, 2.5 pmol E2 conjugating enzyme (combination of 10 different E2s, unless otherwise stated) and 0.6 nmol ubiquitin (Boston Biochemicals, Cambridge, USA) in buffer containing 25 mM Tris–HCl (pH 8.0), 4 mM ATP, 5 mM MgCl_2_, 200 μM CaCl_2_ and 1 mM DTT were incubated in LoBind protein tubes (Eppendorf, Stevenage, UK) for 1 h at 30°C with agitation. Reactions were stopped by the addition of SDS-PAGE sample buffer (25 mM Tris–HCl (pH 6.8), 2.5% β-mercaptoethanol, 1% SDS, 10% glycerol, 1 mM EDTA, 0.05 mg/ml bromophenol blue) and heated for 5 min at 95°C prior to SDS-PAGE and Western blotting.

### Western blotting

Protein extracts (typically 40 μg) or *in vitro* ubiquitylation reactions were separated by 10% Tris-glycine SDS-PAGE and proteins transferred onto an Immobilon FL PVDF membrane (Millipore, Watford, UK). Membranes were blocked using Odyssey blocking buffer (Li-cor Biosciences, Cambridge, UK) and incubated with the primary antibody diluted in Odyssey blocking buffer with 0.1% Tween 20 overnight at 4°C. Membranes were washed with PBS containing 0.1% Tween 20, incubated with either Alexa Fluor 680 or IR Dye 800-conjugated secondary antibodies for 1 h at room temperature and further washed with PBS containing 0.1% Tween 20. Proteins were visualized and quantified using the Odyssey image analysis system (Li-cor Biosciences, Cambridge, UK).

### Clonogenic assays

U2OS cells grown in 10 cm dishes were treated in the absence and presence of TRIM26 or Mule siRNA (200 pmol) using Lipofectamine RNAiMAX (Life Technologies, Paisley, UK) for 48 h. For NEIL1 overexpression, U2OS cells were treated with 500 ng pCMV-Tag3a-NEIL1 mammalian expression plasmid using Lipofectamine 2000 (Life Technologies, Paisley, UK) for 24 h. Cells were trypsinised and a defined number seeded in triplicate into 6-well plates and incubated overnight at 37°C in 5% CO_2_ allowing for the cells to adhere. Note that increasing cell numbers were used for increasing IR doses, and also double the numbers of cells were plated for Mule or TRIM siRNA, to account for cellular plating efficiencies. Cells were then irradiated with up to 4 Gy X-rays. Following treatment, fresh medium was added to the cells and colonies were allowed to grow for 7 days, prior to fixing and staining with 6% glutaraldehyde, 0.5% crystal violet for 30 min. Plates were washed, left to air dry overnight and colonies counted using the GelCount colony analyser (Oxford Optronics, Oxford, UK). Relative colony formation (surviving fraction) was expressed as colonies per treatment level versus colonies that appeared in the untreated control. Statistical analysis was performed using the CFAssay for R package (29).

### Alkaline single cell gel electrophoresis (comet) assay

The comet assay, examining DNA repair activities in-gel, was performed as described (30). Cells were trypsinised, diluted to ∼1 × 10^5^ cells/ml and 250 μl aliquots of the cell suspension placed into the wells of a 24-well plate on ice. The cells were irradiated in suspension and then embedded in 1% low melting agarose (Bio-Rad, Hemel Hempstead, UK) on a microscope slide pre-coated with 1% normal melting point agarose and allowed to set on ice. Cells were allowed to undergo DNA repair in-gel for up to 2 h at 37°C in a humidified chamber, prior to lysis in buffer containing 2.5 M NaCl, 100 mM EDTA, 10 mM Tris–HCl pH 10.5, 1% (v/v) DMSO and 1% (v/v) Triton X-100 for at least 1 h at 4°C. Slides were transferred to an electrophoresis tank and incubated in the dark for 30 min in fresh cold electrophoresis buffer (300 mM NaOH, 1 mM EDTA, 1% (v/v) DMSO, pH 13) to allow the DNA to unwind. The DNA was subjected to electrophoresis at 25 V, 300 mA for 25 min, slides were neutralised with three 5 min washes of 0.5 M Tris–HCl (pH 8.0), and allowed to dry overnight. The slides were rehydrated for 30 min in water (pH 8.0), stained for 30 min with SYBR Gold (Life Technologies, Paisley, UK) diluted 1:20 000 in water (pH 8.0) and allowed to dry prior to imaging. Cells (50 per slide, two slides per time point) were analysed using the Komet 6.0 image analysis software (Andor Technology, Belfast, Northern Ireland) and % tail DNA values averaged from at least three independent experiments.

### E3 ubiquitin ligase identification by tandem mass spectrometry

Strataclean resin (Agilent Technologies, Stockport, UK) was used to extract protein from purified fractions and subsequently resuspended in 0.05% (w/v) Rapigest in 25 mM ammonium bicarbonate. Samples were heated at 80°C for 10 min, reduced with 3 mM DTT at 60°C for 10 min and alkylated in 9 mM iodoacetamide in the dark at room temperature for 30 min. Trypsin (1 μg) in 50 mM acetic acid was added and samples mixed with rotation at 37°C overnight. Digests were acidified by the addition of trifluoroacetic acid (1% (v/v)) and held at 37°C for 45 min prior to centrifugation at 17 200 x g for 30 min. Supernatants were collected and diluted to ∼50 ng/μl in 0.1% (v/v) trifluoroacetic acid (TFA)/3% (v/v) acetonitrile. LC peptide separations were carried out using an Ultimate 3000 nano system (Dionex/Thermo Fisher Scientific, Hemel Hempstead, UK) and for each analysis the sample was loaded onto a trap column (Acclaim PepMap 100, 2 cm × 75 μm inner diameter, C_18_, 3 μm, 100 Å) at 5 μl/min with an aqueous solution containing 0.1% (v/v) TFA and 2% (v/v) acetonitrile. After 3 min, the trap column was set in-line with an analytical column (Easy-Spray PepMap^®^ RSLC 50 cm x 75 μm inner diameter, C_18_, 2 μm, 100 Å). Peptide elution was performed by applying a linear gradient of solvent A (HPLC grade water with 0.1% (v/v) formic acid) and solvent B (HPLC grade acetonitrile 80% (v/v) with 0.1% (v/v) formic acid). Mass spectrometry was performed using the Q Exactive instrument operated in data dependent positive (ESI+) mode to automatically switch between full scan MS and MS/MS acquisition. Survey full scan MS spectra (*m/z* 300–2000) were acquired with 70 000 resolution (*m/*z 200) after accumulation of ions to 1 × 10^6^ target value based on predictive automatic gain control (AGC) values from the previous full scan. Dynamic exclusion was set to 20 s. The 10 most intense multiply charged ions (*z* ≥ 2) were sequentially isolated and fragmented in the octopole collision cell by higher energy collisional dissociation (HCD) with a fixed injection time of 100 ms and 35 000 resolution. Typical mass spectrometric conditions were as follows: spray voltage, 1.9 kV, no sheath or auxillary gas flow; heated capillary temperature, 275°C; normalised HCD collision energy 30%. The MS/MS ion selection threshold was set to 2 × 10^4^ counts. A 2 Da isolation width was set. Raw data files were searched in Mascot against the UniProt human database. A precursor ion tolerance of 10 ppm and a fragment ion tolerance of 0.01 Da were used with carbamidomethyl cysteine set as a fixed modification and oxidation of methionine as a variable modification. The false discovery rate (FDR) against a decoy database was 1%.

### Identification of NEIL1 ubiquitylation sites by tandem mass spectrometry

Following separation of *in vitro* ubiquitylation reactions containing His-NEIL1 and GST-Mule or His-TRIM26 by 10% SDS-PAGE, the region corresponding to monoubiquitylated NEIL1 was identified and excised. The gel pieces were covered with a minimum volume of 25 mM ammonium bicarbonate in 1.5 ml LoBind protein tubes (Eppendorf, Stevenage, UK) and heated at 37°C for 15 min. This was repeated twice more to wash the gel pieces. For the reduction step, 10 mM DTT in 25 mM ammonium bicarbonate was added and the samples incubated at 56°C for 1 h. For alkylation, 55 mM iodoacetamide was added and the sample incubated at 37°C in the dark. The gel pieces were washed with alternate washes of 25 mM ammonium bicarbonate followed by 25 mM ammonium bicarbonate/acetonitrile (2:1 v/v). For protease digestion, ArgC (10 ng/μl in 25 mM ammonium bicarbonate containing 1 mM DTT) was added and incubated on ice for 15 min. 25 mM ammonium bicarbonate was added to cover the gel pieces and samples heated at 37°C overnight. The samples were centrifuged for 15 min at 17 200 × g, the supernatant removed and vacuum centrifuged to near dryness. Acetonitrile (3% v/v) and TFA (0.1% v/v) was added, the samples were centrifuged for 15 min before transfer to a total recovery vial for LC–MS analysis.

## RESULTS

### Purification of the major E3 ubiquitin ligases responsible for ubiquitylating NEIL1

**Figure 1.**
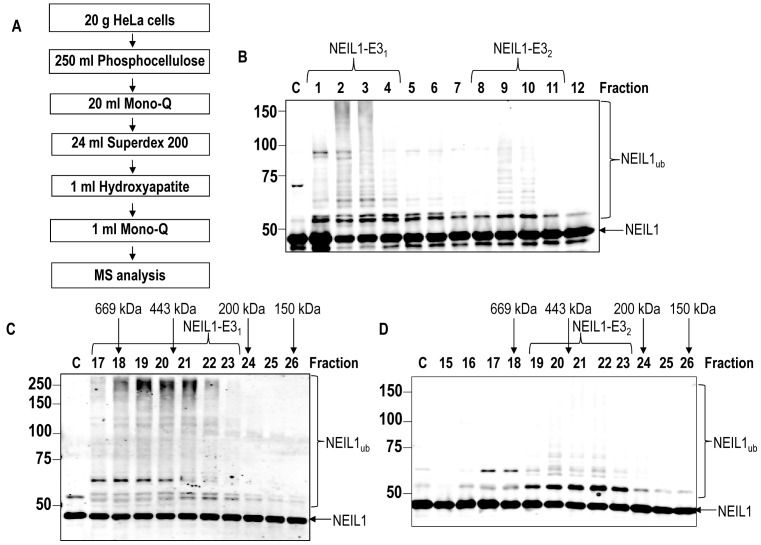
Initial purification of the E3 ubiquitin ligases for NEIL1. (**A**) Scheme for the purification of the E3 ubiquitin ligases for NEIL1 from HeLa cell extracts. (**B**) *In vitro* ubiquitylation of His-tagged NEIL1 by fractions obtained from the first ion exchange (Mono Q) chromatography. (**C** and **D**) *In vitro* ubiquitylation of His-tagged NEIL1 by fractions obtained from size exclusion (Superdex 200) chromatography containing (**C**) NEIL1-E3_1_ or (**D**) NEIL1-E3_2_. Shown above the figures are the positions of elution of known molecular weight standards. In all experiments, *in vitro* ubiquitylation of His-tagged NEIL1 (4.6 pmol) was performed in the presence of E1 activating enzyme (0.7 pmol), ubiquitin (0.6 nmol; Ub) and all E2 conjugating enzymes (2.5 pmol) and analysed by 10% SDS-PAGE and immunoblotting using His-tag antibodies. Molecular weight markers are indicated on the left hand side of appropriate figures and the positions of unmodified and ubiquitylated NEIL1 (NEIL1_ub_) are shown.

**Figure 2.**
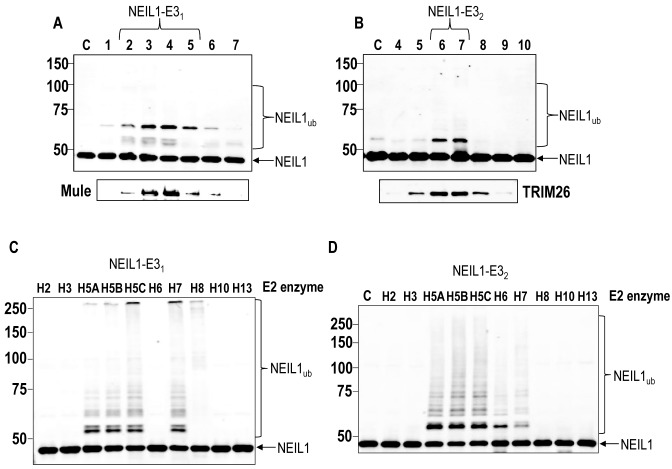
Identification of Mule and TRIM26 as E3 ubiquitin ligases for NEIL1. (**A** and **B**) *In vitro* ubiquitylation of His-tagged NEIL1 by fractions obtained from the final 1 ml Mono Q chromatography containing (A) NEIL1-E3_1_ or (B) NEIL1-E3_2_. Below each figure is the alignment of active fractions with either Mule or TRIM26, respectively as detected by Western blotting. (**C** and **D**) *In vitro* ubiquitylation of His-tagged NEIL1 by an active fraction purified from HeLa whole cell extracts containing either (C) NEIL1-E3_1_ or (D) NEIL1-E3_2_ in the presence of individual E2 conjugating enzymes. In all experiments, *in vitro* ubiquitylation of His-tagged NEIL1 (4.6 pmol) was performed in the presence of E1 activating enzyme (0.7 pmol), ubiquitin (0.6 nmol; Ub) and E2 conjugating enzymes (2.5 pmol) and analysed by 10% SDS-PAGE and immunoblotting using His-tag antibodies. Molecular weight markers are indicated on the left hand side of appropriate figures and the positions of unmodified and ubiquitylated NEIL1 (NEIL1_ub_) are shown.

Since NEIL1 has been demonstrated to be an important enzyme in removal of oxidised DNA bases, particularly in single stranded DNA generated through transcription and replication (12,13), and is therefore important in the cellular response to IR and oxidative stress (15,16), we hypothesized that the protein should be under the control of the UPP catalysed by E3 ubiquitin ligases. Therefore, using an *in vitro* ubiquitylation assay in combination with cell extract fractionation by column chromatography (Figure 1A), an approach which we have used successfully for other BER proteins (28,31,32), we purified the major E3 ubiquitin ligases for NEIL1. Using HeLa cells as a source of protein for fractionation, in combination with recombinant His-tagged NEIL1 as the substrate for *in vitro* ubiquitylation, we were able to obtain protein fractions generated from Phosphocellulose, followed by ion exchange (Mono Q) chromatography that contained two significant ubiquitylation activities against NEIL1 (Figure 1B; see fractions 1–4 and 8–11). These NEIL1-specific ubiquitylation activities (labelled NEIL1-E3_1_ and NEIL1-E3_2_) appeared to be catalysing polyubiquitylation of NEIL1 as revealed by a ladder of protein bands separated by ∼8 kDa protein shifts. These enzymatic activities were purified further separately by size exclusion chromatography (Superdex 200). This revealed that NEIL1-E3_1_ enzymatic activity eluted with a molecular weight of 400–600 kDa in size (Figure 1C; see peak fractions 19–21), whereas NEIL1-E3_2_ eluted at ∼200–400 kDa (Figure 1D; see peak fractions 20–22). These E3 ubiquitin ligase activities were subsequently purified using hydroxyapatite chromatography, and finally by polishing using a second ion exchange (Mono Q) column. At this point, the activities appeared to be predominantly as mono- or diubiquitylation of NEIL1 (Figure 2A, see fractions 2–5 and Figure 2B, see fractions 6–7). The *in vitro* ubiquitylation reactions for initial protein purification containing NEIL1 were performed in the presence of ten different E2 conjugating enzymes. When independent reactions containing separate E2 enzymes were completed, it was discovered that ubiquitylation of NEIL1 by NEIL1-E3_1_ was dependent on the H5 class of E2 enzymes, and H7 (Figure 2C). Similarly, NEIL1-E3_2_ also had a preference for the H5 enzymes, but to a lesser extent, both H6 and H7 in ubiquitylating NEIL1. Highly purified fractions from the final ion exchange (Mono Q) chromatography column were analysed by nanoLC–MS/MS tandem mass spectrometry which revealed that the major E3 ubiquitin ligase in the sample containing NEIL1-E3_1_ was Mcl-1 ubiquitin ligase E3 (Mule; also known as ARF-BP1/HUWE1) with a coverage of 29% (Supplementary Figure S1A). In contrast, in the sample containing NEIL1-E3_2_ the major enzyme was tripartite motif 26 (TRIM26), with a coverage of 64% (Supplementary Figure S1B). When these highly purified proteins fractions were subsequently analysed for the presence of these enzymes by Western blotting, there was a perfect alignment of Mule protein with NEIL1-E3_1_ activity (Figure 2A, see lower panel) and TRIM26 with NEIL1-E3_2_ (Figure 2B, see lower panel). This demonstrates that Mule and TRIM26 are the most likely enzymes catalysing NEIL1 polyubiquitylation in these highly purified fractions generated from human cell extracts.

### Mule and TRIM26 ubiquitylate NEIL1 *in vitro* within C-terminal lysine residues

**Figure 3.**
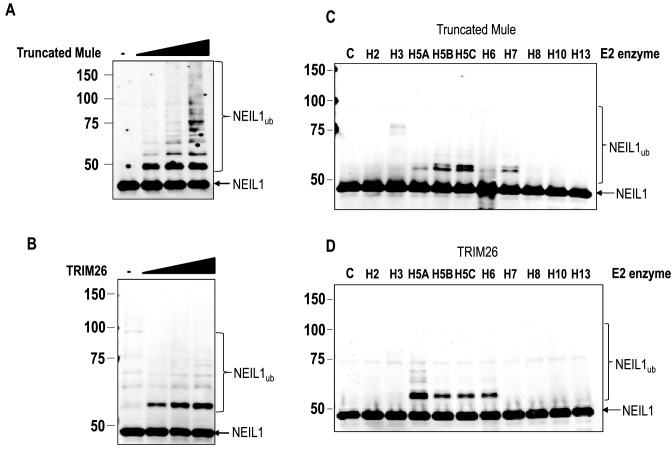
Recombinant truncated Mule and TRIM26 ubiquitylate NEIL1 *in vitro*. (**A–D**) *In vitro* ubiquitylation of His-tagged NEIL1 in the absence and presence of either (**A** and **C**) GST-tagged truncated Mule or (**B** and **D**) His-tagged TRIM26. In all experiments, *in vitro* ubiquitylation of His-tagged NEIL1 (4.6 pmol) was performed in the presence of E1 activating enzyme (0.7 pmol) and ubiquitin (0.6 nmol; Ub). Additionally in (A) the H5c E2 conjugating enzyme (2.5 pmol) was used in the presence of increasing amounts of truncated Mule protein (1.6, 3.1 and 6.3 pmol, respectively). In (B), the H5a E2 conjugating enzyme (2.5 pmol) was used in the presence of increasing amounts of TRIM26 protein (3.1, 6.3 and 12.5 pmol, respectively). In (C and D) individual E2 enzymes (2.5 pmol) were used with either truncated Mule (4.7 pmol) or TRIM26 (12.5 pmol). All reactions were analysed by 10% SDS-PAGE and immunoblotting using His-tag antibodies. Molecular weight markers are indicated on the left hand side of appropriate figures and the positions of unmodified and ubiquitylated NEIL1 (NEIL1_ub_) are shown.

To confirm that TRIM26 is active in ubiquitylating NEIL1 *in vitro*, we cloned full length human TRIM26 into a bacterial expression plasmid and purified the full length protein following overexpression in *Escherichia coli* cells, to a purity of >80% (Supplementary Figure S2A). Similarly, we expressed and purified a truncated Mule containing the E3 ligase active site HECT domain, as previously described, to confirm the *in vitro* ubiquitylation activity of Mule against NEIL1 (28,33) (Supplementary Figure S2B). We were able to confirm that purified, recombinant truncated GST-tagged Mule (Figure 3A) and full length His-tagged TRIM26 (Figure 3B) are capable of substantially polyubiquitylating NEIL1 *in vitro*. We also performed *in vitro* ubiquitylation reactions in the presence of individual E2 conjugating enzymes and confirmed, similar to the protein fractions containing Mule purified from HeLa cell extracts (Figure 2C), that recombinant Mule activity is dependent on the H5 class of enzymes and H7 (Figure 3C). Recombinant TRIM26 activity was also dependent on the H5 class of E2 enzymes and on H6 (Figure 3D), which is similar to purified protein fractions known to contain TRIM26 (Figure 2D). Cumulatively, these results demonstrate that the active E3 ubiquitin ligases targeting NEIL1 for polyubiquitylation purified from human cell extracts, are indeed Mule and TRIM26.

**Figure 4.**
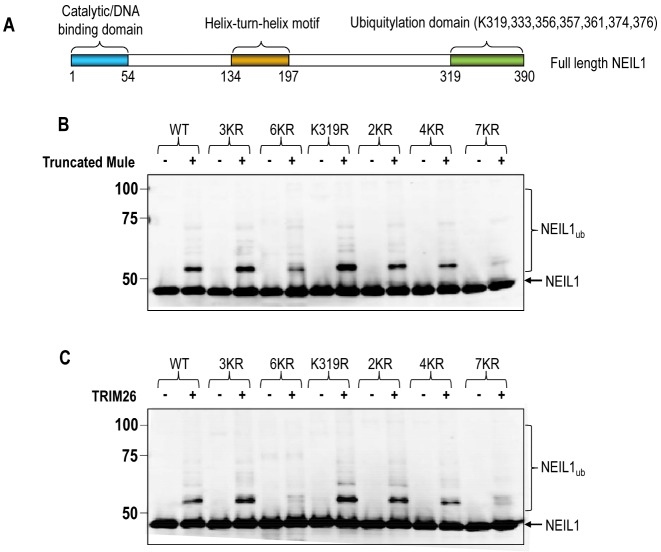
Identification of sites of ubiquitylation within the C-terminus of NEIL1 by recombinant truncated Mule and TRIM26. (**A**) Schematic showing the protein domains within the full length NEIL1 protein. *In vitro* ubiquitylation of His-tagged NEIL1 mutants in the absence and presence of either (**B**) GST-tagged truncated Mule or (**C**) His-tagged TRIM26. In all experiments, *in vitro* ubiquitylation of His-tagged NEIL1 (4.6 pmol) was performed in the presence of E1 activating enzyme (0.7 pmol) and ubiquitin (0.6 nmol; Ub). Additionally in (B) the H5c E2 conjugating enzyme (2.5 pmol) was used in the presence of GST-tagged truncated Mule protein (4.7 pmol) and in (C) the H5a E2 conjugating enzyme (2.5 pmol) was used in the presence of His-tagged TRIM26 protein (12.5 pmol). All reactions were analysed by 10% SDS-PAGE and immunoblotting using His-tag antibodies. Molecular weight markers are indicated on the left hand side of appropriate figures and the positions of unmodified and ubiquitylated NEIL1 (NEIL1_ub_) are shown. For the NEIL1 mutants: 3KR = K333/356/357R; 6KR = K333/356/357/361/374/376R; 2KR = K319/333R; 4KR = K319/333/356/357R; 7KR = K319/333/356/357/361/374/376R.

In order to identify the sites of NEIL1 ubiquitylation by Mule and TRIM26, we initially generated protein truncations of NEIL1 for examination in the *in vitro* ubiquitylation assay. These data indicated that a C-terminal deletion of NEIL1 lacking the final 55 amino acids (see protein schematic in Figure 4A) was unable to be substantially ubiquitylated *in vitro* (data not shown). We then used a mass spectrometry based approach whereby NEIL1 was ubiquitylated *in vitro* using purified recombinant Mule and TRIM26. Proteins were separated by SDS-PAGE, the band corresponding to monoubiquitylated NEIL1 (∼52 kDa) was located, excised, the protein extracted and digested with Arg-C prior to mass spectrometry analysis. This digestion strategy was designed to maximise the peptide coverage of the C-terminal end of NEIL1, which from our C-terminal deletion mutant, appeared to be the major site of ubiquitylation. Whilst total protein coverage was ∼60%, this only covered 7 out of 24 lysines within NEIL1, but crucially captured 4 out of 8 lysines within the C-terminus of the protein (Supplementary Figure S3A). The only lysine residue found to be ubiquitylated using this approach was lysine 319 (K319) (Supplementary Figure S3B). We therefore created a mutant His-NEIL1 containing a lysine to arginine mutation at residue 319 using site directed mutagenesis, and then analysed *in vitro* ubiquitylation of this mutant by truncated Mule and TRIM26. However, and consistent with our previous studies using this approach (28,31), mutation of this single lysine residue was unable to suppress ubiquitylation by Mule (Figure 4B) or TRIM26 (Figure 4C). In fact the K319R NEIL1 mutant appeared to be a better substrate for ubiquitylation than the wild type (WT) protein. We therefore began to mutate lysine residues sequentially, in addition to K319, toward the C-terminus of the protein and started to see a decrease in the efficiency of ubiquitylation, compared to the wild type protein, by both truncated Mule and TRIM26 when four lysine residues (319, 333, 356, 357; 4KR) were mutated. We also observed a substantial decrease in ubiquitylation of a six lysine mutant (333, 356, 357, 361, 374, 376; 6KR) but not with a three lysine mutant (333, 356, 357; 3KR) which both contained lysine 319. However, ubiquitylation was almost completely suppressed by mutation of all seven lysine residues (319, 333, 356, 357, 361, 374, 376) within the immediate C-terminal region of NEIL1. In summary, these data suggest that NEIL1 is ubiquitylated *in vitro* by Mule and TRIM26 on exactly the same seven lysine residues present within the immediate C-terminal end of the protein.

### Mule and TRIM26 regulate cellular steady state levels of NEIL1 via ubiquitylation

**Figure 5.**
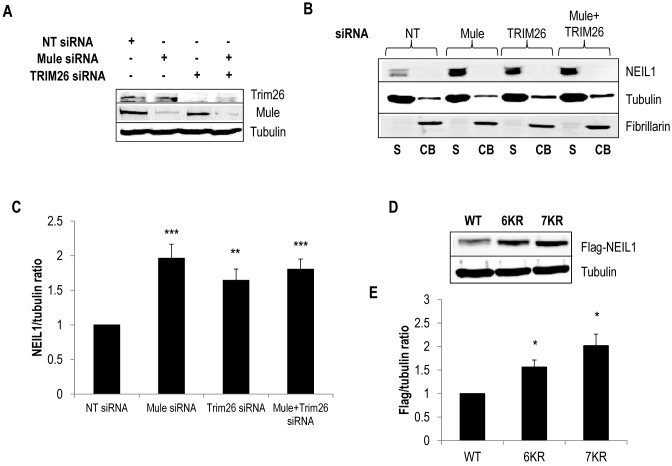
Cellular steady state NEIL1 protein levels are regulated by Mule and TRIM26. (**A–C**) U2OS cells were grown in 10 cm dishes for 24 h to 30–50% confluency and then treated with Lipofectamine RNAiMAX transfection reagent (10 μl) in the presence of 200 pmol non-targeting (NT), Mule and/or TRIM26 siRNA for 72 h. (A) Whole cell extracts were prepared and analysed by 10% SDS-PAGE and immunoblotting with the indicated antibodies. (**B**) Proteins were separated by biochemical fractionation, and the soluble (S) and chromatin bound (CB) fractions analysed by 10% SDS-PAGE and immunoblotting with the indicated antibodies. (C) Levels of NEIL1 protein relative to tubulin in the soluble fraction were quantified from at least three independent experiments. Shown is the mean NEIL1/tubulin ratio with standard errors normalised to the non-targeting (NT) siRNA-treated control which was set to 1.0. (**D**) U2OS cells were grown in 10 cm dishes for 24 h to ∼90% confluency and then treated with Lipofectamine 2000 transfection reagent (10 μl) in the presence of 150 ng mammalian expression plasmids for Flag-tagged wild type (WT) or NEIL1 mutants (6KR = K333/356/357/361/374/376R; 7KR = K319/333/356/357/361/374/ 376R) for 24 h. Whole cell extracts were prepared and analysed by 10% SDS-PAGE and immunoblotting with the indicated antibodies. (**E**) Levels of Flag-tagged NEIL1 proteins relative to tubulin were quantified from at least three independent experiments. Shown is the mean Flag-NEIL1/tubulin ratio with standard errors normalised to the WT-NEIL1 transfected cells which was set to 1.0. *P < 0.05, **P < 0.02, ***P < 0.01 as analysed by a one sample *t*-test.

To determine the cellular role of NEIL1 ubiquitylation by Mule and TRIM26, we examined the effect of siRNA-mediated depletion of these enzymes in cultured U2OS cells. Firstly, we demonstrated that siRNA sequences targeting Mule, TRIM26 or the combination of both are very effective in reducing the corresponding cellular protein levels in comparison to a non-targeting siRNA control (Figure 5A). We then examined NEIL1 protein levels in the absence of these enzymes using biochemical fractionation of cellular proteins from U2OS cells. Interestingly, we discovered that the majority of the NEIL1 protein was extractable in a soluble form (S), and not strongly bound to chromatin (CB) (Figure 5B, compare lanes 1 and 2). NEIL1 also appeared as a doublet, suggesting that either two isoforms are present, or that the protein is post-translationally modified (e.g. by phosphorylation). To confirm this, we observed that both of these protein bands appear to decrease using NEIL1 siRNA (Supplementary Figure S4A). In the absence of Mule or TRIM26, we discovered that protein levels of NEIL1 are significantly elevated versus the non-targeting siRNA control (Figure 5B, compare lanes 1 and 3, or 1 and 5), and that the lower doublet band of NEIL1 was predominantly responsive to these treatments. In fact NEIL1 protein levels increased by nearly ∼2-fold and 1.6-fold following Mule or TRIM26 siRNA, respectively (Figure 5C). These results are consistent with our observations that Mule and TRIM26 are efficient in catalysing polyubiquitylation of NEIL1 *in vitro* (Figures 1B, 2C and D and 3A and B), and that this mechanism contributes to ubiquitylation-dependent degradation of NEIL1 in cultured cells. Interestingly, when a double knockdown of Mule and TRIM26 was performed, there was no further increase in NEIL1 protein levels. This indicates that Mule and TRIM26 target the same population of NEIL1 for ubiquitylation-dependent degradation. To further support this observation, we analysed the cellular protein stability of exogenously expressed NEIL1 mutants lacking either six or seven C-terminal lysine residues (NEIL1 6KR and 7KR), which are unable to be efficiently ubiquitylated *in vitro* by Mule or TRIM26 (Figure 4B and C). Consistent with these *in vitro* data, NEIL1 6KR, and more so NEIL1 7KR, are more stable than the wild type NEIL1 protein following expression in U2OS cells (Figure 5D). In fact protein stability increases by 1.5-fold and 2.2-fold, respectively (Figure 5E). These data confirm that ubiquitylation of NEIL1 within the C-terminal region catalysed by Mule and TRIM26 contributes to degradation of cellular NEIL1.

### The cellular response to IR is differentially regulated by Mule and TRIM26

**Figure 6.**
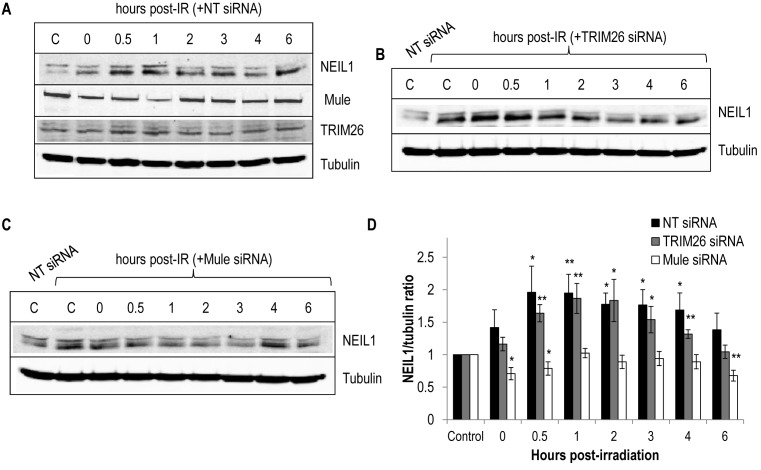
Cellular NEIL protein levels are induced in response to ionising radiation in a Mule-dependent manner. (**A–C**) U2OS cells were grown in 10 cm dishes for 24 h to 30–50% confluency and then treated with Lipofectamine RNAiMAX transfection reagent (10 μl) in (A) presence of non-targeting (NT) siRNA, (**B**) 200 pmol TRIM26 siRNA or (C) 200 pmol Mule siRNA for 72 h. Cells were subsequently irradiated (10 Gy) and harvested at the various time points post-irradiation. Whole cell extracts were prepared and analysed by 10% SDS-PAGE and immunoblotting with the indicated antibodies. C refers to unirradiated control in either the presence of non-targeting (NT), Mule or TRIM26 siRNA. (**D**) Levels of NEIL1 protein relative to tubulin were quantified from at least three independent experiments. Shown is the mean NEIL1/tubulin ratio with standard errors normalised to the unirradiated control which was set to 1.0. *P < 0.05, **P < 0.01 as analysed by a one sample *t*-test of ratios comparing the time points post-irradiation relative to their respective, untreated controls.

Whilst we demonstrated that Mule and TRIM26 are important in controlling steady state protein levels of NEIL1, we extended these observations to analyse whether these enzymes are important in modulating NEIL1 during the cellular DNA damage response. We discovered that NEIL1 protein levels are induced in U2OS cells in the presence of non-targeting (NT) siRNA in response to IR, and reach a peak at ∼1 h post-irradiation where the levels are ∼2-fold higher than that seen in the unirradiated controls (Figure 6A and D). Interestingly, the levels of Mule appear to decrease at similar time points, suggesting that reduced levels of this E3 ubiquitin ligase enzyme are possibly causing a deficiency in ubiquitylation-dependent degradation of NEIL1 and allowing the protein to accumulate. NEIL1 protein levels then start to decrease at 4–6 h post-irradiation, at a time when levels of Mule also recover to control levels. We noted again that the lower band of the NEIL1 doublet appeared to be responsive to changes, whereas the upper band remained relatively constant. To determine whether Mule and/or TRIM26 are directly involved in modulating cellular protein levels of NEIL1 following IR, and above those already elevated due to increased steady state levels (Figure 5B and C), we suppressed the levels of these enzymes by siRNA. Similar to these previous results, we again demonstrate that a knockdown of Mule or TRIM26 causes an elevation in the protein stability of NEIL1 in comparison to non-targeting (NT) siRNA, unirradiated control (Figure 6B and C; compare lanes 1 and 2). In addition, downregulation of TRIM26 had no significant effect on the further induction of NEIL1 protein by IR, in comparison to the non-targeting (NT) siRNA treated cells (Figure 6B and D). In contrast, an siRNA-mediated knockdown of Mule suppressed the further elevation in NEIL1 protein post-irradiation, and in fact protein levels marginally decreased during this time period and appeared to return to those seen in the non-targeting (NT) siRNA, unirradiated control (Figure 6C and D). This clearly demonstrated that the induction of NEIL1 required during the cellular DNA damage response is mediated through a Mule-dependent process.

**Figure 7.**
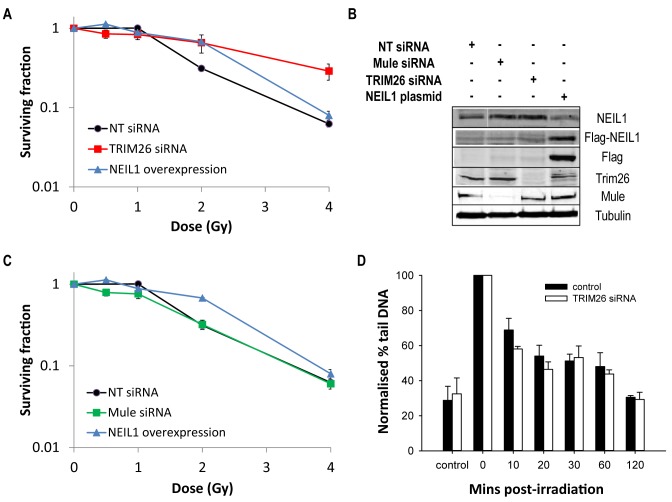
TRIM26 controls cellular sensitivity to IR. (**A–C**) U2OS cells were grown in 10 cm dishes for 24 h to 30–50% confluency and then treated with Lipofectamine RNAiMAX transfection reagent (10 μl) in the presence of 200 pmol non-targeting (NT) siRNA, TRIM26 siRNA or Mule siRNA for 72 h. U2OS cells were also treated with Lipofectamine 2000 transfection reagent (10 μl) in the presence of 500 ng mammalian expression plasmid for NEIL1 (NEIL1 overexpression) for 24 h. (A and C) Clonogenic survival of cells was analysed following treatment with increasing doses of ionising radiation (0–4 Gy). Shown is the surviving fraction with standard error of the mean from at least three independent experiments. (**B**) Whole cell extracts were prepared and analysed by 10% SDS-PAGE and immunoblotting with the indicated antibodies. (**D**) Cells were irradiated (1.5 Gy) and DNA single strand breaks and alkali labile sites measured at various time points post-irradiation by the alkaline single cell gel electrophoresis (comet) assay. Shown is the % tail DNA with standard deviations from at least three independent experiments.

We subsequently analysed whether the further elevation in NEIL1 protein levels in response to IR contributes to the radiosensitivity of cells by clonogenic assays, and the dependence of this on TRIM26 and Mule. It has been previously demonstrated that an siRNA mediated knockdown of NEIL1 sensitises cells to IR (15) and we confirmed these data which were statistically significant (*P* < 0.0001), even though a knockdown efficiency of only 60% was achievable in comparison to a non-targeting (NT) siRNA control (Supplementary Figure S4B). This suggests that the control of NEIL1 protein levels is important in the IR-induced cellular DNA damage response. Following depletion of TRIM26 by siRNA in U2OS cells, we found that this caused a statistically significant increase (*P* < 0.0001) in resistance to IR-induced cell killing than the non-targeting (NT) siRNA control treated cells (Figure 7A). This increase in cellular radioresistance is predictable due to both the induction of NEIL1 steady state levels following TRIM26 siRNA (Figure 5B) combined with an elevation in NEIL1 caused by IR (Figure 6B). To support this, we moderately overexpressed NEIL1 in control cells to mimic the stabilisation in NEIL1 protein levels seen with knockdown of TRIM26 (Figure 7B), and then examined IR-induced cell survival. Indeed, exogenous NEIL1 expression at moderate levels was able to partially recapitulate cellular resistance to IR, and which was statistically significantly different (*P* < 0.002) compared to the non-targeting (NT) siRNA control (Figure 7A). We also examined the radiosensitivity of cells following Mule siRNA and found that this does not alter dramatically, and was not statistically significant, in comparison to non-targeting (NT) siRNA control treated cells (Figure 7B). This is consistent with the lack of induction of NEIL1 in response to IR in the absence of Mule, and ultimately a reduction in NEIL1 protein post-irradiation similar to those seen in the non-targeting (NT) siRNA, unirradiated control (Figure 6C and D). This suggests a threshold level of NEIL1 above which it can modulate cellular radiosensitivity.

Since we demonstrated that a TRIM26 siRNA modulates cellular radiosensitivity, partly by controlling NEIL1 protein levels, we examined whether this was predictably achieved through alterations in DNA damage repair. Therefore, we analysed the repair of IR-induced DNA single strand breaks and alkali labile sites using the alkaline single cell gel electrophoresis (comet) assay. Interestingly, we were unable to find any significant changes in overall DNA damage repair rates when comparing control and TRIM26 siRNA knockdown cells at various times (0–120 min) post-irradiation (Figure 7D). This suggests that changes in NEIL1 protein levels caused by a depletion of TRIM26, do not contribute greatly to the general repair of DNA damage (e.g. oxidised DNA bases and AP sites) induced by IR. Instead this implies that NEIL1 is involved in the repair of specific DNA damage substrates, which may be greatly contributing to cell death following IR treatment.

## DISCUSSION

The integrity of the genome is under constant threat as a consequence of DNA damage induced by cellular oxidative metabolism, and by exogenous stress including IR. NEIL1 plays an important role in suppressing the accumulation of DNA damage, and thus in maintaining genome stability, by repairing oxidised DNA base damage. Indeed, this is highlighted by observations that NEIL1 protects cells from hydrogen peroxide and IR-induced cell death (15,16). Furthermore, a deficiency of NEIL1 in knockout mouse models displays an obese phenotype associated with a metabolic syndrome (17,18), and loss of NEIL1 has been associated with the earliest symptoms of age-related neurodegenerative disorders such as Alzheimer's and Parkinson's diseases in humans (20,21). We now provide evidence that NEIL1 protein levels are effectively controlled by the UPP. By biochemical fractionation of human cell extracts, in combination with an *in vitro* ubiquitylation assay using NEIL1 as a substrate, we purified and identified two E3 ubiquitin ligases, Mule and TRIM26, which are the major enzymes targeting NEIL1 for polyubiquitylation. We subsequently demonstrated that both enzymes target the same lysine residues present within the C-terminal end of the protein, and that a mutant NEIL1 lacking these lysine residues is more stable in cells. Similarly, an siRNA-mediated knockdown of Mule or TRIM26 in cells causes an elevation in the protein levels of NEIL1, indicating that these enzymes regulate the steady state levels of NEIL1. We also demonstrate that NEIL1 protein levels are induced in response to IR in a Mule-dependent manner.

TRIM26 is a member of the tripartite motif proteins, which are a family (>70 members) of predicted E3 ubiquitin ligase enzymes due to the presence of a RING finger motif (34). There is growing evidence that these enzymes play key roles in cell biology, particularly during innate antiviral immunity. To our knowledge, there are limited data to date suggesting that purified and recombinant TRIM26 has *in vitro* E3 ubiquitin ligase activity against a specific substrate, and that *in vivo* TRIM26 promotes ubiquitylation-dependent degradation of specific cellular targets which is important for a specific cellular process. One limited study suggested ubiquitylation activity against the histone code reader PHF20 following manipulation of TRIM26 in mammalian cells, although this is open to interpretation (35). More convincingly, cellular TRIM26 was demonstrated to promote ubiquitylation-dependent degradation of the transcription factor IRF3 and to negatively regulate interferon-β production and antiviral responses (36). It was shown that viral infection can induce TRIM26 expression, and that by modulating IRF3 and interferon-β this acts as a mechanism to evade the innate immune system. Additionally, we now provide the first evidence that purified, recombinant TRIM26 can directly ubiquitylate NEIL1 *in vitro*, and further modulates the protein levels of NEIL1 *in vivo*. This suggests that TRIM26 may have multiple cellular roles not only in antiviral immunity as proposed for a number of TRIM family members, but also in regulating a key BER enzyme involved in cellular DNA damage repair. Indeed these roles of TRIM26 may be associated, as there is accumulating evidence that the cellular DNA damage response mediated through several DNA repair pathways is directly linked with innate immunity (37,38). Interestingly, it has recently been described that TRIM26 functions as a novel tumour suppressor of hepatocellular carcinoma, and that an siRNA knockdown of TRIM26 promotes cell proliferation and metastasis (39). We also demonstrated that an absence of TRIM26 significantly increases cellular resistance to IR, partially through elevating NEIL1 protein levels. Cumulatively, these data highlight the importance of TRIM26 in controlling cellular processes required for the maintenance of genome stability, and in prevention of human disease development such as cancer.

In addition to TRIM26, we also identified Mule as a second E3 ubiquitin ligase involved in controlling cellular steady state protein levels of NEIL1. We demonstrated that Mule and TRIM26 appear to target the same pool of NEIL1 for ubiquitylation-dependent degradation, since an siRNA knockdown of both enzymes did not cause an additive effect. These E3 ubiquitin ligases also target the same lysine residues on NEIL1 for ubiquitylation. Mule has previously been shown to ubiquitylate and control the levels of a number of BER proteins, including Pol β (28), DNA polymerase λ (Pol λ) (40), MutYH (41), but also targets the p53 tumour suppressor protein (42) and Mcl-1 (43). It therefore appears to play a central role in the cellular DNA damage response by controlling not only DNA repair protein levels, but also proteins involved in cell proliferation and apoptosis. Indeed we have previously demonstrated that an siRNA knockdown of Mule causes an acceleration in the kinetics of repair of oxidative DNA damage, as visualised by the comet assay (28). Whilst we demonstrated this was mainly due to Pol β misregulation (as evidenced by no changes in DNA repair kinetics in Pol β^−/−^ versus Pol β^+/+^ cells) our new data and those mentioned above would suggest that the cellular effect of Mule on DNA repair appears to be multi-faceted. Indeed, Mule-dependent regulation of BER occurs both at the level of damaged DNA base excision (NEIL1 and MutYH) and during DNA gap filling (Pol β and λ).

Interestingly we also show that the induction of NEIL1 protein following IR is Mule-dependent, but is not affected by the absence of TRIM26. *NEIL1* mRNA levels have previously been shown to be induced in response to oxidative stress (44,45). However, this is the first demonstration for the upregulation of NEIL1 protein in response to DNA damage by a post-translational modification event, as the increase in protein is initiated immediately post-irradiation. Whilst NEIL1 induction following IR is obviously not caused by a direct effect of Mule on NEIL1, it does suggest that Mule activity and/or presence is indirectly co-ordinating the IR-induced elevation in NEIL1 protein. Intriguingly, we observed that cellular NEIL1 appeared as a doublet following SDS-PAGE and Western blotting, with the lower band being specifically responsive to any changes caused by IR or modulation through Mule or TRIM26. This raises the possibility that NEIL1 is further regulated by other post-translational modifications, such as phosphorylation or acetylation, and that a depletion of Mule imbalances the regulation of NEIL1 required for the cellular response to DNA damage. Indeed, BER proteins in general have been shown to be controlled by numerous post-translational modifications (24), although the mechanism specifically related to Mule-dependent regulation of NEIL1 requires further investigation. Nevertheless, we have shown that a Mule knockdown does not alter cellular radiosensitivity, as whilst the steady state levels of NEIL1 are increased in the absence of Mule, post-irradiation NEIL1 protein levels seem to decrease similar to those seen in the non-targeting siRNA treated, unirradiated controls. The lack of change in radiosensitivity is surprising in itself given that Mule regulates a number of cellular proteins, as described above, for ubiquitylation-dependent degradation. This result though, is in contrast to a TRIM26 knockdown where an increase in both steady state, and IR-induced levels of NEIL1 are associated with cellular radioresistance.

Why the steady state levels of NEIL1 appears to be under the control of two E3 ubiquitin ligases is currently unclear, but ultimately suggests that cellular NEIL1 protein must be tightly regulated. The specific cellular role of NEIL1 is still under debate as whilst the enzyme was thought to be a backup enzyme for the major oxidative DNA glycosylases OGG1 and NTH1 when it was first isolated, there are more recent reports suggesting that it may be involved in co-ordinating the repair of specific DNA damage substrates. For example, NEIL1 has been proposed to remove base damage from single stranded DNA and bubble structures (12,13), suggesting a specific role for the enzyme during transcription and replication, and this is further supported by evidence that NEIL1 associates with DNA replication proteins (14). NEIL1 can also act on telomeric DNA (9,10). Consistent with these data, we demonstrate in this manuscript that an elevation in NEIL1 protein caused by a TRIM26 knockdown does not impact on overall DNA damage repair kinetics, as revealed by the comet assay, immediately following IR. However a knockdown of TRIM26, or a partial NEIL1 overexpression, does mediate resistance of cells to IR. This is consistent with previously published data demonstrating that mouse embryonic cells lacking NEIL1 are hypersensitive to IR and hydrogen peroxide-induced cell death (15,16). As we revealed cellular resistance by clonogenic assays, in which colonies are allowed to grow for ∼7 days post-irradiation, this would suggest that NEIL1 acts on specific DNA damage substrates (e.g. during replication and transcription) which are more efficiently repaired by elevated NEIL1 protein levels caused by the depletion of TRIM26. However, further studies are required to truly understand the preferred substrates and role of NEIL1 in the cellular DNA damage response. Nevertheless in summary, we have now demonstrated that cellular NEIL1 is regulated by the UPP mediated by the E3 ubiquitin ligases Mule and TRIM26, which plays a vital role in co-ordinating the cellular response to DNA damage.
